# Tips and tricks for hysteroscopic resection of a large type I submucosal fibroid: a video presentation

**DOI:** 10.1093/jscr/rjag055

**Published:** 2026-02-12

**Authors:** Alexandros Fotiou, Andrew Heck, Raj Mathur, Nikolaos Tsampras

**Affiliations:** Department of Obstetrics and Gynecology, Saint Mary’s Hospital, Manchester University NHS Foundation Trust, Oxford Road, Manchester M13 9WL, United Kingdom; Department of Obstetrics and Gynecology, Saint Mary’s Hospital, Manchester University NHS Foundation Trust, Oxford Road, Manchester M13 9WL, United Kingdom; Developmental Biology and Medicine, School of Medical Sciences, The University of Manchester, Manchester, United Kingdom; Developmental Biology and Medicine, School of Medical Sciences, The University of Manchester, Manchester, United Kingdom

**Keywords:** hysteroscopic myomectomy, submucosal leiomyoma, bipolar resectoscope, operative hysteroscopy

## Abstract

We present a step-by-step approach for hysteroscopic myomectomy in patients with submucosal fibroids, focusing on key surgical techniques that enhance safety, preserve uterine integrity, and ensure complete fibroid removal. A 49-year-old woman with heavy menstrual bleeding presented with a type I submucosal fibroid measuring 32 × 23 × 20 mm. The procedure was performed using a 27 Fr bipolar resectoscope. Intraoperative strategies include: minimal cervical dilation to reduce fluid leakage, continuous saline infusion with controlled suction for optimal visibility, avoidance of repeated scope removal to maintain intrauterine pressure and identification of myometrial margins to ensure complete resection and prevent perforation. This video highlights essential surgical techniques and intraoperative considerations for performing safe and effective hysteroscopic myomectomy. Attention to fluid management, visualization, and preservation of cervical and uterine integrity is critical for optimal outcomes, especially in women with prior uterine surgery or fertility concerns.

## Introduction

Uterine fibroids represent the most frequently occurring benign tumors of the female reproductive tract. Their prevalence among women of reproductive age varies significantly, reported from 4.5% to 68.6% [1]. Prevalence is influenced by differences in population demographics and the diagnostic methods employed.

It is estimated that at least 50% of women with uterine fibroids remain asymptomatic. However, this proportion is likely underestimated, as asymptomatic cases often remain undiagnosed and unreported. However, uterine fibroids can cause considerable morbidity including menorrhagia, or prolonged bleeding, which may result in iron deficiency anemia. In addition, large fibroids can produce pain or pelvic pressure symptoms including increased urinary frequency or urinary incontinence. Subfertility and recurrent miscarriage may also arise, especially when submucosal myomas distort the uterine cavity [2].

Furthermore, uterine fibroids have a significant financial impact on affected women and the national healthcare system. A systematic review concluded that uterine fibroids may impose costs of up to 34.4 billion USD per year in the United States [3]. Management of uterine fibroids depends on the presence and severity of symptoms, the type of uterine fibroid based on the classification of the International Federation of Gynecology and Obstetrics, as well as patient preferences and fertility considerations [4].

Hysteroscopic resection of uterine fibroids is safe and effective surgical treatment for submucosal fibroids. The procedure preserves uterine integrity, maintaining fertility, and is associated with high rates of symptom improvement and patient satisfaction.

The aim of this video article is to outline practical tips and techniques for hysteroscopic myomectomies, with a focus on fertility preservation and successful conception.

## Case presentation (patients and methods)

A 49-year-old woman was referred to our outpatient hysteroscopy clinic for evaluation and management of heavy menstrual bleeding requiring blood transfusions. Her obstetric history was notable for two previous lower segment cesarean sections.

Initial management prior to referral involved a 3-month course of a gonadotropin-releasing hormone analog, leuprorelin, aiming to reduce the severity of uterine bleeding. Pelvic imaging, including both transabdominal and transvaginal ultrasound, demonstrated a heterogeneous myometrium and identified a submucosal fibroid measuring 32 × 23 × 20 mm.

An outpatient hysteroscopy revealed a type I submucosal fibroid with a maximal diameter exceeding 3 cm. Due to its size and intracavitary location, hysteroscopic myomectomy under general anesthesia was deemed the most appropriate treatment approach. The patient’s history of previous cesarean sections was also taken into consideration, as it may increase the risk of uterine perforation during the procedure.

Although the patient had completed her family, she expressed a strong preference for hysteroscopic resection over laparoscopic hysterectomy due to the anticipated shorter recovery time. She was also eager to discontinue medical therapy owing to the emergence of menopausal symptoms.

## Intraoperative tips and tricks (discussion)

For hysteroscopic myomectomy, we used a bipolar resectoscope (Olympus OES Pro, 27 French). Cervical dilation was kept to a minimum (Hegar dilator 7 mm) to avoid excessive fluid leakage through the cervix, which can compromise intrauterine distention and visualization.

Optimal fluid management is essential for procedural safety and clear operative field. When not employing a continuous fluid management system, it is key that the team has a mechanism for continuous fluid deficit monitoring, including the anesthetist. The procedure was paused after each 3-liter bag of saline to calculate fluid balance and continued only if the fluid deficit remained within safe limits. In our case, the total fluid deficit was 400 mL, well below the maximum safe threshold of 2500 mL, as recommended by the British Society for Gynecological Endoscopy guidelines [5]. Continuous saline infusion was balanced with selective suction, which was applied at the surgeon’s discretion to restore visibility when the view became obscured. The suction device was connected directly to the hysteroscope, allowing dynamic regulation of intrauterine pressure and clarity.

To maintain cervical integrity and preserve distention, we avoided withdrawing the resectoscope to remove tissue chips during the procedure. Although floating chippings can reduce visibility, repeated instrument removal may lead to excessive cervical dilatation, further fluid leakage and difficulty to distend the vacity.

Ensuring complete fibroid resection is crucial for symptom resolution and to minimize the risk of recurrence or the need for repeat surgery. Clear identification of the myometrial margins is emphasized to confirm resection completeness while avoiding uterine perforation. The myometrium appears red-pink and is relatively softer comparing to the pale and firm fibroid.

The procedure lasted approximately 20 minutes.

In this video we demonstrate key surgical steps, techniques, and intraoperative tips for performing hysteroscopic myomectomy.

## Conclusion

The patient recovered well with minimal postoperative vaginal bleeding and was fit to be discharged the same day as the procedure. The patient’s periods returned to normal after the procedure and she remained asymptomatic in her three months’ follow up.

In summary, this case highlights useful tips and tricks to perform a hysteroscopic myomectomy. Minimal cervical dilatation, constant awareness of accurate fluid balance, dynamic use of saline suction, and clear identification of the myometrial margins are key factors for a successful procedure.

## Data Availability

The dataset used is available from the corresponding author on reasonable request.

## Transparency

The authors confirm that this manuscript represents a truthful, precise, and transparent description of the study conducted. No significant details have been omitted, and any variations from the initial study protocol have been appropriately disclosed and justified.

Video Link: https://drive.google.com/file/d/15tY7vP4PhsOwoa7K9owKJnQILTqhTrfQ/view
